# Two complicated cases of severe scrub typhus, eschar- a non-negligible sign: Case reports and literature review

**DOI:** 10.1097/MD.0000000000039879

**Published:** 2024-09-27

**Authors:** De-Han Cai, Xiao-Lin Fang

**Affiliations:** a Nephrology Department in Jiangxi Provincial People’s Hospital, the First Affiliated Hospital to Nanchang Medical College, Nanchang, China; b Department II of Respiratory and Critical Care in Jiangxi Provincial People’s Hospital, the First Affiliated Hospital to Nanchang Medical College, Nanchang, China.

**Keywords:** early organ damage, eschar, MODS, *O tsutsugamushi*, scrub typhus

## Abstract

**Rationale:**

Scrub typhus is a mite-borne, acute febrile disease caused by *Orientia tsutsugamushi*. The endemic areas of scrub typhus are expanding, both globally and in China. Patients who are not treated promptly, are likely to die of multiple organ dysfunction syndrome.

**Patient concerns:**

Case I A 61-year-old female patient complained of sudden chest tightness and shortness of breath accompanied by fever for 6 days. Case II A 54-year-old male patient complained of fever and cough for 4 days and renal insufficiency for 2 days.

**Diagnoses:**

Scrub typhus, multiple organ dysfunction syndrome.

**Interventions:**

After the definite diagnosis, both patients were treated with doxycycline and various organ supports.

**Outcomes:**

The patient in case I was ultimately not salvageable. The patient in case II was successfully cured by the prompt administration of doxycycline along with continuous renal replacement therapy.

**Lessons:**

With early diagnosis and treatment, patients can completely recover. Eschar, a characteristic sign of scrub typhus, is often overlooked, leading to delayed diagnosis and regrettable outcomes.

## 1. Introduction

Scrub typhus, which is transmitted by the bites of *Leptotrombidium* mites, is an acute infectious disease caused by *Orientia tsutsugamushi*. Their hosts are primarily rodents. Pathogens and their associated vectors (mites) lack thermostatic mechanisms, making many aspects of their biology (e.g., reproduction and survival rates) highly vulnerable to climate change.^[1]^

Scrub typhus is a serious public health issue in the Asia Pacific region. It threatens 1 billion people worldwide and causes 1 million illnesses annually. The traditional endemic area for scrub typhus is known as the “scrub typhus triangle.” It covers an area of more than 8 million km^2^, from the Russian Far East in the north to Pakistan in the west, Australia in the south, and Japan in the east.^[2]^

The pathogen reproduces from the bite site of *Leptotrombidium* mites, forms skin lesions, and enters the blood circulation via the lymphatic system, causing vasculitis and perivasculitis, leading to congestion and edema of tissues and organs, cell degeneration, and necrosis, eventually resulting in varying degrees of organ function damage.^[3]^ Without appropriate treatment, the disease has a mortality rate of up to 70%.^[2]^ The following are 2 cases of complicated severe scrub typhus at our hospital. It is hoped that this will deepen physicians’ understanding of the disease and prevent catastrophic outcomes.

## 2. Case presentation

**Figure 1.**
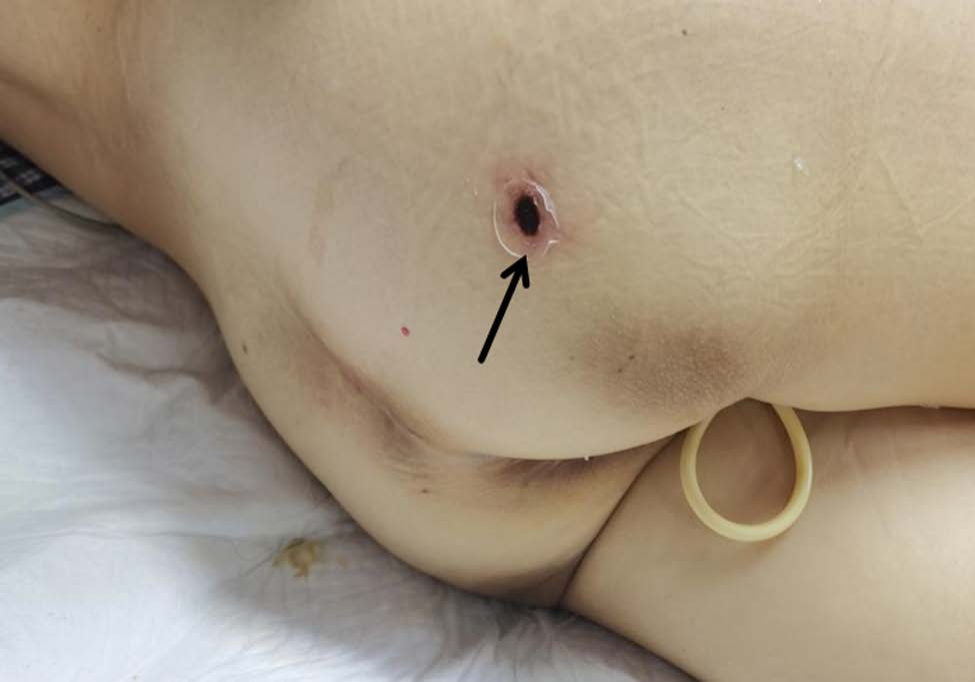
An eschar was seen on the right buttock of the patient in case I.

**Figure 2.**
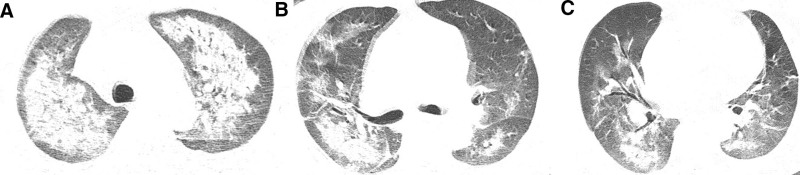
Chest CT of the patients in case I. (A–C) Patchy infiltrating shadows in both lungs are symmetrically distributed along the bronchovascular bundles.

Case I A 61-year-old housewife from Nancheng County, Fuzhou City, Jiangxi Province, was admitted to the Cardiovascular Medicine Department of our hospital on September 22, 2023, with sudden chest tightness and shortness of breath accompanied by fever for 6 days. The patient had no obvious cough, sputum, chest pain, hemoptysis, abdominal pain, or diarrhea. The patient was transferred to our hospital after the treatment at a local hospital was ineffective. Vital signs on admission were as follows: temperature, 38.2°C; respiratory rate 33 beats/min; heart rate, 114 beats/min; and blood pressure, 106/63 mm Hg. She was conscious, with a 2 × 3 cm lesion on the skin of the buttock (Fig. 1). More wet rales were heard in both lungs. The heart rhythm was regular with no murmurs. Marked edema was observed in both lower extremities. The laboratory results are presented in Table 1. The patient’s human immunodeficiency virus, treponema pallidum antibody, antineutrophil cytoplasmic antibodies, and rheumatoid immunity series were all negative. Chest computed tomography (CT) images are shown in Figure 2A–C.

**Table 1:** Laboratory findings of the 2 patients on admission to our hospital in both cases.

Test items	*WBC(×10^9^/L)	*NE (%)	HB (g/L)	*PLT (×10^9^/L)	*CRP (mg/L)	*PCT (ng/mL)	*ALB (g/L)	*TB (μmol/L)	*DB (μmol/L)	*GPT (IU/L)	*GOT(IU/L)	*CREA(μmol/L)	*LDH(IU/L)	*NT-ProBNP (pg/mL)	*PT (sec)	*PT-INR	*APTT (sec)	*D-Dimmer (mg/L)	†pH	†PaCO_2_ (mm Hg)	†PaO_2_ (mm Hg)	†Lac (mmol/L)	FERR (ng/mL)
Case I	15.5	63.1	81	32	>170	3.24	23.2	75.3	59.8	82	240	169	802	13,430	15.2	1.22	49.4	2.89	7.362	28.7	57.3	7.3	>2000
Case II	9.91	88.1	136	24	152	7.34	22.4	47.1	37.2	77	116	1144	673	316	14.9	1.05	27.6	14.59	7.35	34	71	3.1	>2000
Normal range	3.5–9.5	40–75	115–150	125–350	0–10	0–0.05	40–55	3.42–20.5	0–6.84	7–40	13–35	41–81	120–250	204.4	9.4–12.5	0.8–1.2	25.4–38.4	0–0.24	7.35–7.45	35–48	83–108	0.5–2.0	13–150

ALB = albumin, APTT = activated partial thromboplastin time, CREA = creatinine, FERR = ferritin, GOT = glutamic oxaloacetic transaminase, GPT = glutamic pyruvic transaminase, HB = hemoglobin, Lac = lactic acid, LDH = lactate dehydrogenase, NE = neutrophil, PaCO_2_ = arterial blood carbon dioxide partial pressure, PaO_2_ = arterial blood oxygen partial pressure, PCT = procalcitonin, pH = potential hydrogen, PLT = platelets, PT-INR = prothrombin time-international normalized ratio, WBC = white blood cells.

* All specimens are peripheral blood.

† The indicators from pH to Lac are arterial blood gas analysis.

The patient developed shock and oliguria on the day of admission. Bedside ultrasonography revealed a hyperdynamic cardiac state with collapsed inferior vena cava. The shock at this point was considered to be the result of a combination of infection, heart failure, and hypovolemia. Appropriate fluid replacement was administered to expand the blood volume. Dopamine and dobutamine were given to raise blood pressure and cardiac strength. Meanwhile, recombinant human brain natriuretic peptide was used. However, the patient’s blood pressure still failed to reach the value of the lower limit of the normal range after more than 1 hour. Subsequently, norepinephrine was added. Piperacillin Tazobactam was given for anti-infection. Due to severe hypoproteinaemia, the patient was given 50 mL albumin infusion. Polyene phosphatidylcholine, magnesium isoglycyrrhizinate, and ademetionine 1,4-butanedisulfonate were administered for hepatoprotection, enzyme, and bilirubin lowering. However, on the same day, the patient’s condition quickly progressed from hypoxemia to acute respiratory distress syndrome. Endotracheal intubation and ventilator-assisted ventilation were performed. The antibiotic regimen was adjusted to vancomycin combined with imipenem cilastatin.

Bedside bronchoscopy performed on September 23 revealed significant airway mucosal edema with no purulent secretions or bleeding. Fungi or bacteria were not detected in bronchoalveolar lavage fluid cultures. On September 24, a repeat routine blood test showed that the hemoglobin had dropped to 64 g/L, and the coagulation review showed that the prothrombin time was 15.8 seconds, the prothrombin time-International Normalized Ratio was 1.4, and the activated partial thromboplastin time was 67.9 seconds, hence the plasma was transfused. On September 25, a repeat routine blood test showed that platelets had dropped to 12 × 10^9^/L. Although there was no obvious sign of bleeding, emergency platelet transfusion was performed, and recombinant human thrombopoietin was used to correct coagulation function. Except for improved coagulation, the patient had progressive decreases in hemoglobin and platelets, and progressive increases in liver enzymes, bilirubin, procalcitonin, and lactate dehydrogenase.

After careful examination and discussion on September 25, the experts of the multidisciplinary consultation concluded that the skin lesion on the patient’s right buttock was eschar (Fig. 1). Scrub typhus was highly suspected combined with the clinical manifestations. It was advised to use doxycycline first and perform blood next-generation sequencing (NGS). As doxycycline injections were not available in our hospital, oral formulations were used. To salvage the patient, we contacted the pharmacy department to request temporary procurement of doxycycline IV preparations, so the patient was started on doxycycline IV preparations on September 26. On the same day, because of anuria and further increase in creatinine levels, continuous renal replacement therapy was administered. On September 27, NGS revealed that the causative pathogen was *O tsutsugamushi*. Unfortunately, the patient’s family decided to discontinue further treatment, and the patient died the day after discharge.

Case II A 54-year-old male farmer from Dexing City, Shangrao District, Jiangxi Province, was admitted to the Nephrology Department of our hospital on October 22, 2023, with fever and cough for 4 days and renal insufficiency for 2 days. The patient had a temperature of up to 39.1°C, accompanied by dry cough and shortness of breath, with no abdominal pain or diarrhea. The patient was transferred to our hospital when his creatinine rose to 1044 µmol/L. Vital signs on admission to our hospital were as follows: temperature, 36°C; heart rate, 112 beats/min; respiratory rate, 20 beats/min; and blood pressure, 118/60 mm Hg. The patient was conscious and a rash was visible on the face. Respiratory sounds were clear in both lungs, with no dry or wet rales. The heart rhythm was regular with no murmurs. Edema was not observed in both lower extremities. The laboratory results are presented in Table 1. The patient tested negative for human immunodeficiency virus, treponema pallidum antibodies, rheumatologic series, antineutrophil cytoplasmic antibodies series, and antiglomerular basement membrane antibodies. Abdominal ultrasound showed a slightly swollen volume in both kidneys and slightly enhanced echogenicity of both renal parenchyma. Chest CT showed scattered striated foci in both lungs with bilateral pleural effusions.

The patient was treated with piperacillin-tazobactam for antiinfection, human interleukin-11 for platelet elevation, and magnesium isoglycyrrhizinate for hepatoprotection, hemodialysis, and albumin infusion. Based on the hematologist opinion platelet transfusion were withheld as ADAMTS13 estimation was not available in our hospital that could have ruled out thrombotic thrombocytopenic purpura. Monitoring of changes in ferritin, triglycerides, fibrinogen, and blood counts was recommended for the timely detection of hemophagocytic syndrome. The patient’s fragmented red blood cells were <1%, and platelet transfusion was administered when the patient’s platelet count decreased to 4 × 10^9^/L.

**Figure 3.**
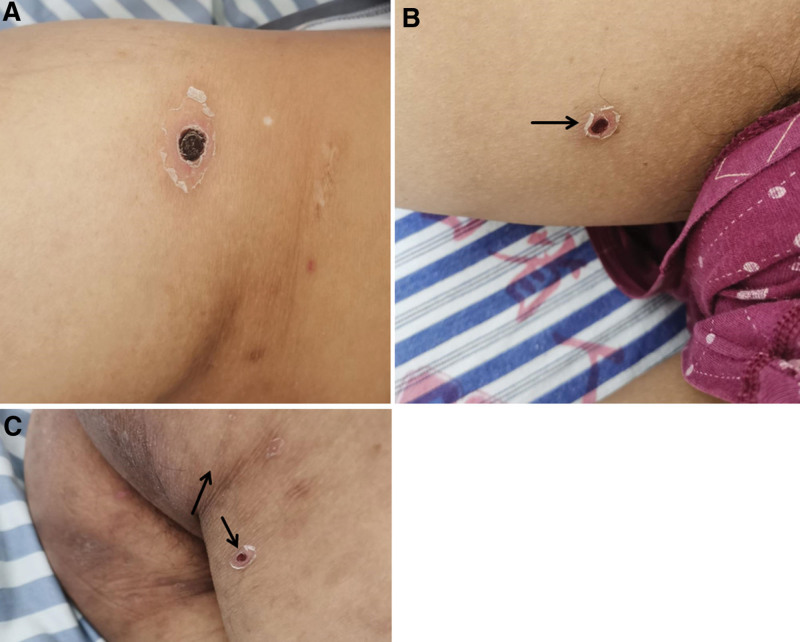
Multiple eshcars on the patient in case II. (A) An eschar was seen in the right groin of patient in case II. (B) An eschar was seen on the ventral sides of the right thigh root of patient in case II. (C) An eschar was seen on the dorsal sides of the right thigh root of of patient in case II.

On October 25, the supervising physician’s examination revealed that the patient had a round brown eschar with a diameter of 5 mm under the left eyelid, and similar skin lesions were seen on the right thigh and waist (Fig. 3A–C). On the same day, doxycycline was administered for antiscrub typhus treatment and blood NGS was performed. On October 26, the patient’s temperature decreased. On October 27, the temperature was completely normalized and NGS revealed the result for *O tsutsugamushi*. On October 28, the patient’s cough, sputum, and shortness of breath improved, urine output began to increase, scleral yellowing subsided, and platelets counts rebounded. The patient was cured and discharged 17 days after the hospitalization.

## 3. Discussion

Scrub typhus is a life-threatening acute febrile disease caused by *O tsutsugamushi*. The antigenic heterogeneity of *O tsutsugamushi* and transient immunity in the body after infection lead to massive primary infections and reinfections. Scrub typhus is now appearing as a reemerging infectious disease. The drivers of the transmission and reemergence of scrub typhus include, but are not limited to, globalization, climate change, urbanization, and human expansion into previously uninhabited areas.^[4]^

Located in southeastern China, our province has a subtropical monsoon climate, abundant rainfall, favorable climate, and hilly terrain, which are suitable for the breeding of vector organisms. In southern China, it occurs mainly during the summer. In northern China, it is more likely to spread in the fall and winter.^[5]^ Until 2014, cases of scrub typhus were reported in all provinces in China except Qinghai Province.^[4]^ Reports published by countries in the traditional “scrub typhus triangle” indicate that scrub typhus cases have increased over the past decade. Recent evidence from the Arabian Peninsula, Chile, and Kenya suggests that this disease has a wider global distribution in tropical and subtropical areas. Therefore, it is essential to improve physician awareness of scrub typhus.

Eschar is a typical clinical feature of scrub typhus. Both of the presented cases had eschars. The patient in case II had multiple eschars and one on the face, an uncommon site, which was rare. The larvae of chiggers usually feed on thin, tender, or wrinkled skin.^[2]^ They prefer to invade moist, strong-smelling, and hidden parts of the body. Eschars are often found in the groin, perianal area, perineum, external genitalia, armpits, and occasionally on the chest, breasts, umbilicus, toes, eyelids, etc.^[6]^ It starts as a pimple, then ulcerates, and forms a black scab, like skin burned by cigarettes. Most systemic symptoms of the disease appear in the second week,^[7]^ and eschar occurs before the appearance of these symptoms.^[2]^ Therefore, the detection of eschar is important for early diagnosis of scrub typhus.

The larvae of chiggers do not puncture the skin of the host; therefore, the patient’s pain is not noticeable. As long as there is no secondary infection, there is no exudation around the eschars, the patient is neither painful nor itchy. In addition, the darker skin color in some patients makes the eschars less noticeable. There are several possible reasons for physicians to ignore eschars: inadequate knowledge of scrub typhus and eschars, lack of careful examination, and different strains of *O tsutsugamushi* having different abilities to induce eschars, resulting in the atypical appearance of eschars.^[8]^ Lymph nodes near eschars are often enlarged, mobile, painful, and nonpurulent. Physicians can use enlarged lymph nodes to search for nearby eschars.^[6]^

One thing to state is that eschar has great diagnostic cue value, but the presence or absence of eschar does not confirm or deny the diagnosis of scrub typhus. Because not every diagnosed patient develops eschar. The percentage of patients with scrub typhus who develop eschars ranges from 1% to 97% depending on ethnicity. Caucasians and East Asians are more likely to present with eschars than South Asians are. Additionally, eschar is not exclusive to scrub typhus. Eschar can also be found in patients with spotted fever, anthrax, spider bites, and leishmaniasis.^[2]^

Serologic and/or molecular testing, such as enzyme-linked immunosorbent assay, immunofluorescence assay, immunochromatographic testing, Weil–Felix test, and polymerase chain reaction, is essential to confirm the diagnosis of scrub typhus. The specificity and sensitivity of the Weil–Felix test are low because of the use of nonrickettsial antigens. Immunofluorescence assay and enzyme-linked immunosorbent assay are the most commonly used serological tests, but these methods cannot identify the infection at an early stage and require sampling during the recovery period. Polymerase chain reaction has gradually been accepted for its higher sensitivity and specificity for early diagnosis.^[9]^ Both cases were definitively diagnosed by NGS, which is also a rapid and accurate test for diagnosing scrub typhus.

In addition to recognizing eschar and using tests such as serology, it is equally important to recognize severe scrub typhus. Patients with an Acute Physiology and Chronic Health Evaluation II score of ≥10, which was measured within 24 hours of initial admission, were regarded as severe cases.^[10]^ Severe scrub typhus is associated with substantial complications and death.^[11]^ Severe complication was defined as a new onset of problems and conditions as below: central nervous system: altered consciousness, convulsions, cerebral hemorrhage, or cerebral infarction; respiratory system: chest X-ray or CT showing infiltration of both lungs with at least one of the following conditions: oxygenation index ≤250 mm Hg, respiratory rate ≥30 breaths/min, or mechanical ventilation; cardiac events: myocarditis, myocardial ischemia, or new onset of arrhythmia; renal damage: creatinine ≥177 μmol/L; infectious shock: systolic blood pressure <90 mm Hg, or >40 mm Hg decrease from basal value, except for other causes; gastrointestinal tract bleeding (without peptic ulcer basis).^[10]^ The essence of multiple organ damage (MOD) that occurred in scrub typhus is that the disease can lead to disseminated vasculitis and perivasculitis. MODS is an important cause of severe illness and death in patients with scrub typhus.

Our national study showed that the complication rate of MODS in scrub typhus was 10.12%.^[8]^ Sufficient attention should be paid to these data. It has been stated that age >54 years, disease duration >4.5 days before antibiotics against *O tsutsugamushi*, platelet count < 100 × 10^9^/L, enlarged lymph nodes, skin lesion, lymphocyte percentage >40%, total bilirubin >24 µmol/L, direct bilirubin >8.6 µmol/L, and concomitant underlying diseases are independent risk factors for severe scrub typhus; glutamic pyruvic transaminase >40 IU/L, glutamic oxaloacetic transaminase  >37 IU/L, blood creatinine, and procalcitonin are not independent risk factors for severe scrub typhus.^[12]^ As surrogates of capillary permeability, decreased of platelet counts correlate with disease severity. A multicenter study showed that dyspnea and elevated total bilirubin are the most important predictors in elderly patients with severe scrub typhus.^[13]^

Patients in both cases had several risk factors, such as disease duration >4.5 days before effective treatment, decreased PLT counts, and elevated bilirubin. In addition, the first patient had diffuse infiltration in both lungs. Early identification of the risk factors for severe scrub typhus can help prevent its progression to MODS and reduce mortality.

When a patient first presents with organ failure as a prominent clinical manifestation, physicians tend to focus on the part and ignore the whole behind it, although scrub typhus is known to cause organ damage throughout the body. The most common cause of acute fever is infectious disease.^[14]^ The possibility of infection with a unique pathogen can be inferred from the patient’s rapid onset of severe and complex organ damage. When managing patients with fever, skin lesions, and one or MOD, it is necessary to consider scrub typhus, but also to differentiate between dengue fever, epidemic hemorrhagic fever, sepsis due to special bacteria, and other rickettsiosis. If the first patient had been diagnosed with scrub typhus earlier, the outcome would likely have been different.

Early treatment is only possible with the early diagnosis of scrub typhus and identification of risk factors for severe scrub typhus. Early treatment results in a shorter disease duration and fewer deaths.^[2]^ Rajapakse et al emphasized the importance of initiating appropriate antibiotics when scrub typhus is highly suspected.^[6]^ Antibiotics such as doxycycline, azithromycin, and chloramphenicol are the preferred drugs for the treatment of scrub typhus.^[5]^ Mildly ill patients respond well to oral doxycycline or azithromycin therapy.^[15]^ Combination therapy with intravenous doxycycline and azithromycin is a better therapeutic option for the treatment of severe scrub typhus than monotherapy with either drug.^[11]^

Scrub typhus has different antigenic strains in different endemic countries/regions, and even different strains in the same region. Antigenic variability between strains coupled with the weak and short duration of cross-protection between different strains hampers the development of effective vaccines.^[2]^ Immunoglobulin G is a marker of previous *O tsutsugamushi* infection. One study analyzed cross-sectional immunoglobulin G seroprevalence data and found that the population previously suffered from scrub typhus was substantially underestimated.^[16]^ This group of patients is at risk of reinfection. In this context, how can scrub typhus be effectively prevented and treated?

The 3 links involved in the transmission of infectious diseases must be addressed. First, the rats should be exterminated and environmental sanitation improved. Second, weeding, using insecticides, and chemically treating the soil to eliminate breeding grounds of chiggers. Third, personal precautions should be taken. Avoiding contact with grasslands, woodlands, and other areas of heavy vegetation. People who need to work or perform outdoor activities should apply insect repellents before going outside and wear long clothes and pants, boots, and hats to minimize exposure. Thoroughly clean skin and clothing after working or traveling in a high-risk area.^[17]^ These preventive measures should be publicized and implemented for people who work or live in high-risk endemic areas for scrub typhus.

In conclusion, physicians should be familiar with dynamic changes in endemic areas. Careful physical examination and recognition of eschar, identification of risk factors for severe scrub typhus, and evaluation of early organ damage, are key points in diagnosing scrub typhus. Patients with a high suspicion of scrub typhus and a predisposition to severe illness may be preemptively treated with antibiotics that cover *O tsutsugamushi* to avoid catastrophic outcomes. Prevention and treatment of the disease require concerted efforts by physicians, patients, and government agencies.

## Author contributions

X‐LF conceived the study, put forward valuable opinions, and edited the manuscript; D‐HC produced the draft of the manuscript; all authors have read and approved the manuscript.
